# “Landpartie 2.0” – Conceptual development and implementation of a longitudinal priority program to promote family medicine in rural areas

**DOI:** 10.3205/zma001322

**Published:** 2020-04-15

**Authors:** Linda Seeger, Nadja Becker, Gisela Ravens-Taeuber, Monika Sennekamp, Ferdinand M. Gerlach

**Affiliations:** 1 Goethe University Frankfurt am Main, Institute of General Practice, Frankfurt/Main, Germany

**Keywords:** family medicine, medical students, curriculum, rural health program, shortage of family doctors

## Abstract

**Objective: **This article reports on the conceptual development and subsequent implementation of a targeted and attractive general practice teaching program in a rural area for students of human medicine at the medical faculty of Goethe University, Frankfurt am Main.

**Project description:** Since the 2016/2017 winter semester, usually up to 15 interested students a year have had the opportunity to participate in the longitudinal priority program “Landpartie 2.0”. The program runs for six semesters during the clinical stage of medical studies and consists of regular internships during which the students receive one-to-one support in a family practice, and participate in a series of seminars and an annual one-day excursion. The aim is to enable students, early on in their studies and without any obligations, to gain uninterrupted experience of providing patient care, and to find out what it means to pursue a career in family medicine.

**Results: **Since the beginning of the annual program, 62 students have been included in it. The initial results show that the different elements of the program fulfil the expectations and requirements of participants and that their overall level of satisfaction is high. Almost 95% of students reported that they felt their knowledge had increased as a result of the internships, and they rated them as a useful part of their medical education. Despite the rural focus of the program, around half of the participants were of urban origin.

**Conclusion:** The “Landpartie 2.0” provides students with the opportunity to familiarize themselves with family health care in a rural area. Further studies should investigate to what extent the program encourages students to continue their training with a view to pursuing a career in family medicine.

## Zusammenfassung

**Zielsetzung: **Dieser Beitrag berichtet über die konzeptionelle Entwicklung und anschließende Implementierung eines zielgruppenspezifischen und attraktiven allgemeinmedizinischen Lehrangebots im ländlichen Raum für Studierende der Humanmedizin am Fachbereich Medizin der Goethe-Universität, Frankfurt am Main.

**Methodik: **Seit dem Wintersemester 2016/2017 können in der Regel bis zu 15 interessierte Studierende pro Jahr an dem longitudinalen Schwerpunktprogramm der „Landpartie 2.0“ teilnehmen. Das für den klinischen Studienabschnitt konzipierte, longitudinal über sechs Semester reichende Angebot besteht aus regelmäßigen Praxisphasen in 1:1-Betreuung, einer Seminarreihe und einem jährlichen Tagesausflug. Ziel ist das frühzeitige und kontinuierliche Erleben der Patientenversorgung, um so die eigenen Kompetenzen zu stärken und einen möglichen allgemeinmedizinischen Karriereweg anzuregen.

**Ergebnisse: **Seit dem Start des jährlichen Angebots konnten 62 Studierende in das Programm aufgenommen werden. Erste Evaluationsergebnisse zeigen, dass die Angebotsbestandteile die Erwartungen und Anforderungen der Teilnehmenden erfüllen und eine hohe Programmzufriedenheit besteht. Knapp 95 % der Studierenden berichten von einem subjektiven Wissenszuwachs durch die Praxisphasen und stufen diese als nützlichen Abschnitt ihrer ärztlichen Ausbildung ein. Trotz des ländlichen Fokus des Programms, stammt etwa die Hälfte aller Teilnehmenden aus urbaneren Regionen.

**Schlussfolgerung: **Die „Landpartie 2.0“ stellt eine Möglichkeit dar, Studierende frühzeitig mit der hausärztlichen Versorgung im ländlichen Raum in Kontakt zu bringen. Inwieweit auf diese Weise der weitere Ausbildungs- und Berufsweg mit dem Ziel einer hausärztlichen Tätigkeit positiv beeinflusst wird, soll in Verbleibstudien evaluiert werden.

## Background

It is possible to establish and reinforce an interest in general practice by strongly supporting the subject during medical studies [1], [2]. Priority programs in general practice can contribute towards ensuring that enough young doctors choose to enter the profession. This is particularly relevant in view of the shortage of new recruits that has been the subject of discussion for many years. 

Many such programs have been available outside Germany for decades [3], [4], [5], and a similar development has recently begun in Germany as well [6], [7], [8]. The results of preliminary evaluations have shown that participation in such a program can have a positive influence on preferences in favor of specialization in general practice [9] and possibly pursuing a career in the field [10]. 

Following this development, Goethe University in Frankfurt am Main has worked on a needs- and student-oriented priority program for the promotion of family health care in rural areas. 

A multistage process was chosen in the planning phase, starting with an international literature search according to the snowball principle. Numerous and diverse programs were identified, most of which were in North America and Australia [3], [4], [5]. The accompanying analyses, which included long-term studies, demonstrated a positive effect on the choice of a later career as a rural physician [11]. However, entrance requirements for medical studies, as well as curricula and examination procedures, differed, sometimes considerably, from the German system, making it difficult to adopt them without changes. For this reason, we will focus in the following on existing developments and conditions in Germany. 

In addition to the literature search, we contacted local experts that are interested in supporting young general practitioners in rural areas [12], [13], and discussed how to organize a priority program, and what should be the content of such a program. 

The focus of most of the conceptual development phase was on the courses and overall conditions existing at German universities. As no systematic overview was available of the efforts that German universities were already making to strengthen general practice in rural areas, we conducted a survey of all medical faculties in Germany [6]. In addition, students at Goethe University Frankfurt were asked what they expected of such a priority program [14]. 

### Survey of all medical faculties in Germany

Between July and November 2015, all 37 general practice teaching institutes in Germany were asked what programs they have, or are planning to introduce, to support family medicine in rural areas. The authors received feedback from 31 of the institutes (response rate 83.8%). Twelve of the faculties had 13 relevant programs that focused on supporting family medicine during medical studies. Three of them lasted at least two semesters. Attributes shared by most of the programs were that the number of places available per year was limited and the programs had only recently been introduced. It was noteworthy that unlike the program we were planning, none of the identified priority programs explicitly focused on supporting family medicine in rural areas [6].

#### Student survey at the medical faculty of Goethe University

In order to help ensure the program was well accepted by the target group, we also asked students what they would want and require of a priority program by means of an online survey in summer 2015 [15]. Of the students, 617 in their fourth preclinical semester and later (response rate 28.7%) took part. The results showed that interest in a possible program was considerable, whereby students attached most importance to a program that was practice-oriented, i.e. that included experiencing and actively working in a family practice. They also felt it was important to get to know what running a practice meant from an administrative and commercial perspective [14]. 

The online survey enabled us to gather information and suggestions relating to the organization and content of the individual elements of the program. For example, it was specified that the teaching course should start during the clinical stage of studies, and that besides the continuous practice orientation that is the central element of the program, the series of seminars should include subjects relating to running and managing a practice. 

Using the described multistage development procedure, the priority program “Landpartie 2.0” (“rural outing 2.0”) was developed on the basis of the “Landpartie 1.0”^1^ that has existed since 2012. This paper presents the concept and preliminary evaluation results. 

## Project description

Based on the development stages presented above, the longitudinal six-semester (three-year) “Landpartie 2.0” teaching program was developed between April 2015 and September 2016. 

**Figure 1:**
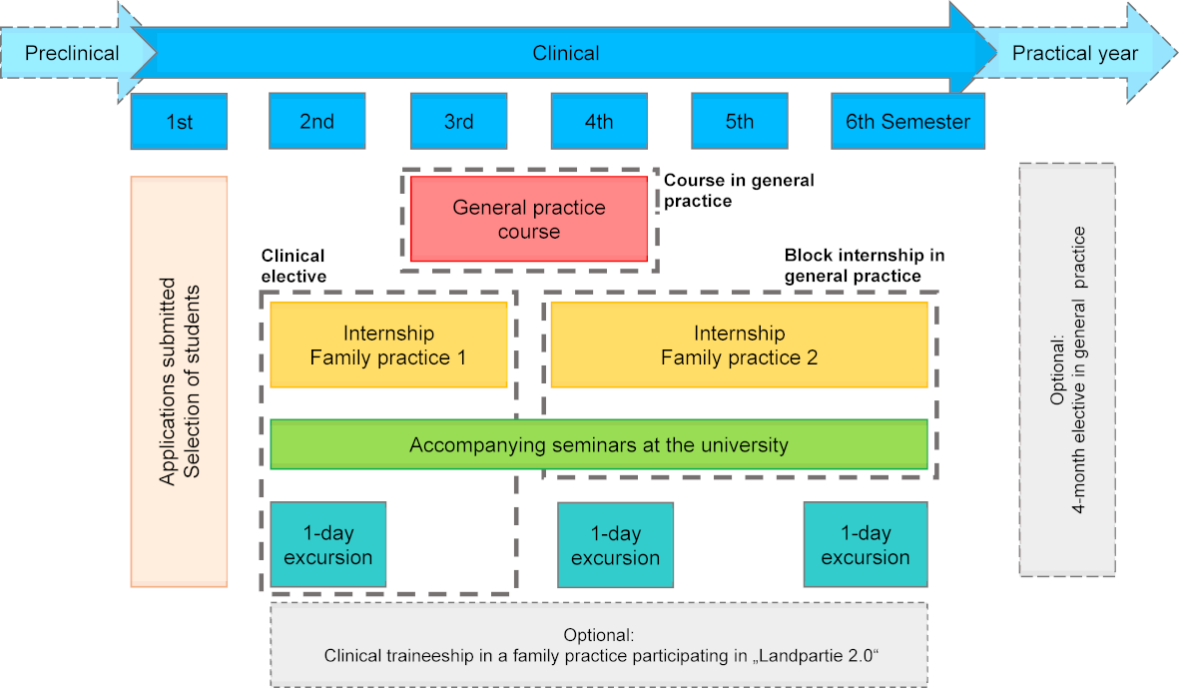
Curriculum timeline of "Landpartie 2.0" in clinical stage of medical studies.

The optional teaching program is directed at students in their first and, in exceptional circumstances, third clinical semesters. In order to apply, the students must complete a self-developed questionnaire and prepare a letter of motivation. As well as requiring socio-demographic details, and information on the careers the students currently foresee for themselves, the application documents also include questions on students’ personal attitudes towards family medicine. Some of the questions are closed and some contain free-text fields, so that applicants have room to individualize their responses, while ensuring manageable comparisons remain possible. Completed questionnaires are then rated according to a fixed point system. The selection of participants takes place on the basis of predictors that correlate positively with a later decision to pursue a career in family medicine [16] and/or to work in a rural area [17], [18]. For example, the probability of being accepted in the program is higher when applicants say they have grown up in a rural region, and are particularly interested in developing long-term relationships to patients or treating a wide range of illnesses in their later day-to-day work. Overall, up to 15 students can generally be included in the program each year, meaning that in three annual cohorts, an average of 45 students can be taking part in the program at any one time. After the recruitment of the students, the program begins with a kick-off event that serves to introduce them to the content and organization of the program and enables all the participants to get to know one another. From the second clinical semester onwards. The practical part of “Landpartie 2.0” begins, which lasts until the end of the clinical phase of studies (see figure 1 (Fig. 1)). 

One of the most important criteria in the development of “Landpartie 2.0” was that the students’ workload should only increase marginally. For this reason, existing compulsory courses and the time allocated to them were incorporated into a new organisational structure and were partly restructured contentwise. By participating in the program, the students complete the obligatory “General practice course” as well as the complete “Clinical elective” and the “Block internship”, but simply in a new and restructured form. In addition, students have the free option to take advantage of organizational support and help in the procurement of clinical traineeships as well as placement in a general practice during the four-month elective in the practical year. 

Regular practical experience with one-to-one support in rural family practices is at the core of the reorganization. The practices are located in one of the three participating districts – Bergstrasse, Hochtaunus and Fulda. Depending on semester, the students spend two to five days (per semester) at their allocated practice. Practice internships, on the other hand, always take place during semester breaks, with the exact period being decided upon by the students and the teaching physician. 

Most of the teaching physicians were recruited especially for “Landpartie 2.0”. When selecting the practices, we ensured that they not only fulfilled standard requirements for academic teaching practices, but were also located in rural communities^2^. After inclusion in the program, all the new teaching physicians are introduced to the organization of the program and undergo one-time training in didactics. Overall, it was possible to recruit 20 teaching physicians, practicing in communities with an average population of 10,238 [range: 2,430-25,345, status as of 30.9.2019], for the “Landpartie 2.0” program. 

In order to familiarize the students with the wide range of tasks that make up family health care, they get to know two practices during five semesters, of which one might be a single-handed practice in a community of 20,000 inhabitants and the other a joint practice in a community of 2,700. The costs of travel and accommodation are covered by the participating districts, and the students receive a catering allowance. 

Parallel to the practice internships, the Institute of General Practice provides an accompanying seminar, covering a wide range of topics ranging from case reviews, a personal exchange of information, strategies used to facilitate consultations (e.g. “breaking bad news”) to the discussion of strategies related to specific therapies. Interdisciplinary subjects (e.g. “Outpatient Palliative Care” and “Psychosomatic Medicine for Outpatients”), as well as the commercial/organizational aspects involved in running a practice (e.g. “Opportunities in the Family Practice – Registration and Remuneration”) are also covered in the series of seminars. The specific topics depend on students’ needs and complement previous study content. Most of the lecturers come from the field of family medicine, with a few coming from the public health sciences. 

For participants in “Landpartie 2.0”, the “General Practice Course” takes place in one of the teaching practices in the rural area and in particularly small groups with a maximum of seven students (generally 8-10). The duration of the course is 10.5 hours and takes place in either the third or fourth clinical semester. Amongst other topics, the students learn about typical procedures and disease patterns that they are likely to be confronted with in a family practice.

Once a year, participants in the program also have the opportunity to go on a one-day excursion to get to know innovative healthcare models. On this occasion, they are introduced to a broad spectrum of different approaches to organizing and collaborating in health care. The destinations of the excursions can be physician and health networks, joint practices, medical care centers and health centers. Participation in one excursion during the three-year period is compulsory. 

Furthermore, in order to support the students and teaching physicians, a logbook is provided, which serves as both a documentation aid throughout the whole program, as well as a basis for organizing the content of internships. The logbook provides the opportunity to stipulate learning objectives for individual internships and thus delivers an overview of the development of individual skills. The main areas of expertise include “Medical Interview Strategies”, “Physical Examination”, “The Use of Diagnostic Tools” and “Therapy/Prevention”. The development of learning objectives is based to a significant extent on those that are used in the standard family medicine internship and that have been tried and tested during medical studies over the long term [19]. The logbook also contains feedback sheets, attendance sheets for all compulsory courses, and assessment sheets for grading students. 

Besides reimbursement for travel and accommodation costs, the students also receive a book voucher and financial support for participation in scientific congresses linked to general practice. 

## Results

Using a number of methodological steps in its development, the priority program “Landpartie 2.0” was successfully designed and implemented and embeds family medicine in the clinical phase of medical studies both longitudinally and with a practical orientation. 

During the winter semester 2016/17, the “Landpartie 2.0” was offered to medical students for the first time. Applications were generally possible in the first clinical semester and the initial number of annual participants was 15. In the first three years, however, the number of applications from students in their first clinical semester was lower than the number of available places. Project management therefore decided to offer the program to students in their third clinical semester as well. However, as they were more advanced in their studies, they only completed the second phase of the internship in a family practice and only some of the program seminars. In this manner, it was possible to fill 16, 15 and 14 of the available places. In the winter semester of 2019/20, the number of applicants was 26 and thus greater than the number of available places for the first time. In response to the number of applicants and the capacity situation, it was therefore decided that two additional students should be allocated a place on the basis of the selection criteria. Since the winter semester 2016/17, it has thus been possible to include 62 students in the program. 

Up to now, the participants have differed considerably from one another in terms of socio-demographic characteristics (see table 1 (Tab. 1)). The ratio of women (69.4%) to men (30.6%) reflects the situation in medical studies overall, but has nonetheless varied from year to year. The average age of participants at the time of application was 24 years (range 20-48), and, as expected, older applicants tended to already have completed vocational training of some kind. On the other hand, it was surprising that around half of the students had come from communities with more than 10,000 inhabitants and thus, according to the definition used in the program, had not grown up in a rural area. This result is in line with free-text responses provided on the application forms. While some applicants repeatedly expressed having a personal connection to the country, other students explicitly mentioned that despite having been raised in a large town, they wanted to take advantage of the opportunity to get to know a different lifestyle and another way of practicing family medicine. 

The preliminary evaluation is of the time spent in the family practices, the accompanying seminars and of the annual one-day excursions, up to the end of the summer semester 2019. Over the long term, a graduate destination survey will also be used find out what actually influenced the decision of students to later work as a family practitioner (in a rural area), and their overall career paths. 

**Figure 2:**
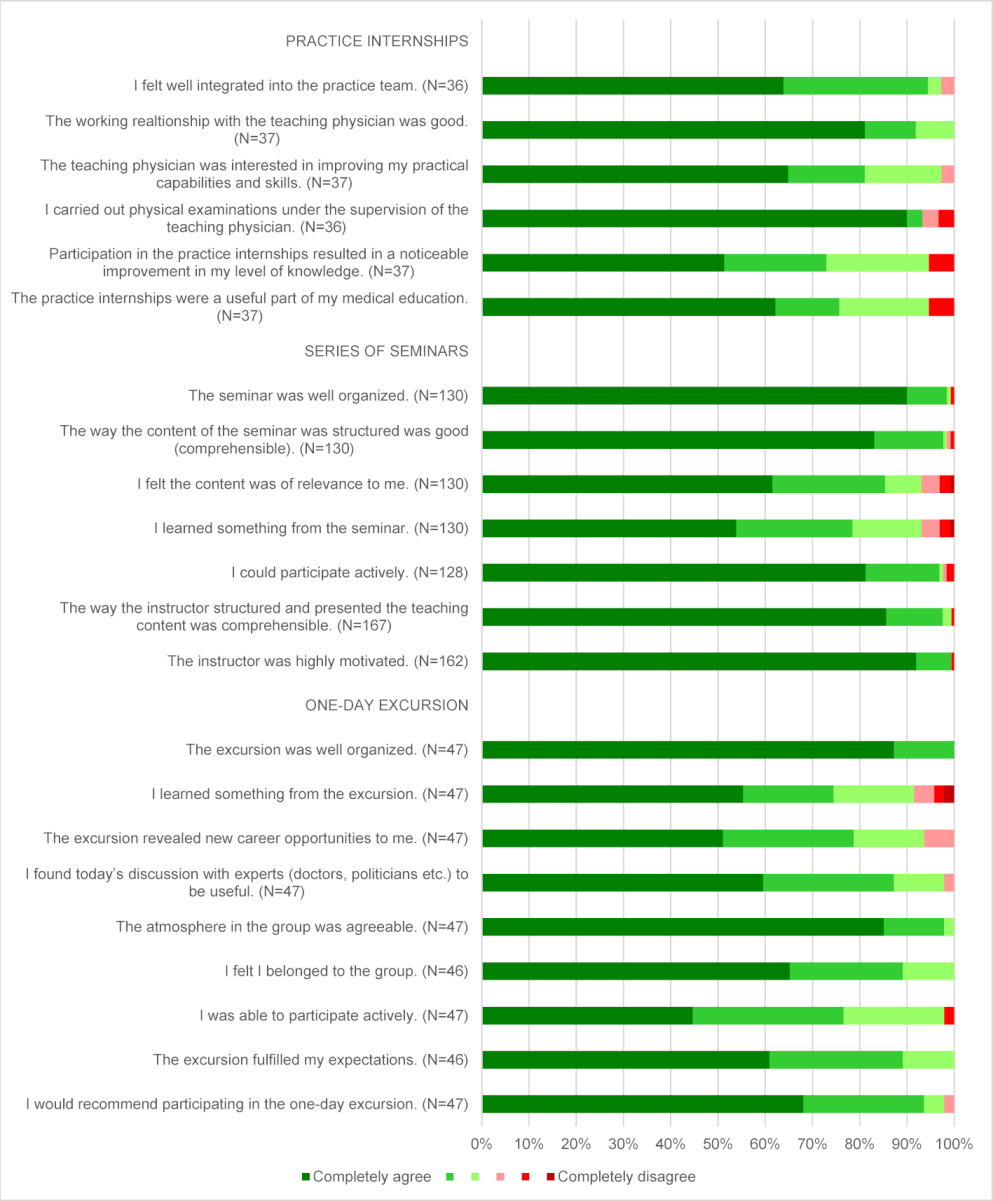
Evaluation results of the practice internships, series of seminars and the one-day excursion up to the summer semester 2019 (Source: proprietary presentation)

All participants completed a two- to five-day internship in the family practices to which they were allocated once per semester, or twice a year. The students had to have attended each family practice at least twice before it was possible to switch. The evaluation then took place per practice and not per internship. Figure 2 (Fig. 2) shows the results from the first two years of the program. It shows that the level of satisfaction was very high with regard to the practical elements of the program. Almost 95% of participants also reported that they felt their level of knowledge had increased and that they considered the practical internships to be a useful part of their medical education. Overall, the internships were allocated the school grade 1.51 (n=37).

Two two-hour seminars take place per semester. The instructors and their number (between one and three instructors per seminar) vary depending on subject. Figure 2 (Fig. 2) shows that the demands that students had of the organization and content of the seminars were almost completely fulfilled, with 93% of students confirming that the content of the seminars was relevant and that their level of knowledge had risen as a result. The instructors were also unanimously considered to demonstrate high levels of motivation and to present the teaching material in a structured manner. 

Since the beginning of “Landpartie 2.0”, three one-day excursions to get to know different innovative models of healthcare have taken place in one of the participating districts. One excursion, for example, was to Ärztehaus Weilrod in Hochtaunuskreis. Six family practitioners work together in the healthcare center, which is made up of four single-handed practices and one group practice. Starting with a self-initiated quality circle that later developed into the organization of a local emergency service, the physicians now work closely with further specialist physicians in the region [20]. Excursion destinations such as Ärztehaus Weilrod provide the participants with an insight into a broad spectrum of physician and interdisciplinary cooperation models. The results of the evaluation show that students’ expectations were consistently met (see figure 2 (Fig. 2)). The organization also received their approval, with 91% of students saying the excursion had contributed towards increasing their level of knowledge, while 94% felt they had gained knowledge of different professional opportunities. Overall, 98% would recommend the excursion to others. The average school grade allocated to it was 1.43 (n=47).

## Discussion

Using a broad-based methodological approach, it was possible to conceptually develop the “Landpartie 2.0” priority program between 2015 and 2016 and to implement the changes from 2016/2017 onwards. Since the beginning of the teaching program, 62 interested students have been motivated to take part in it. 

At its core, the “Landpartie 2.0” consists of regular internships at selected and trained rural practices in the Hessian districts of Bergstrasse, Fulda and Hochtaunuskreis. The internships are accompanied by seminars, the “General Practice Course” in small groups, and an annual one-day excursion to become acquainted with innovative heath care models. In order to prevent the students’ workload from increasing, or to at least confine any increase to a minimum, the students that participated in the program were automatically considered to have completed the obligatory “Clinical elective” and “Block internship in General Practice”. 

As recommended by medical experts and students [21], [22], program participation leads to an increase in the practical share of students’ education in general practice. The objective is that by experiencing intense contact with patients early on, participants should develop and strengthen their practical, communication and social skills. The academic teaching physicians serve the students as role models and mentors, both in seminars and in the practices, and are thus able to strengthen student interest in family medicine [23]. 

The voluntary character of the program enables participants to get to know both family health care and what it means to work in a rural area without having to commit themselves over the long term, as they would if they set up in private practice in one of the three participating districts. Recurring and early contact with family health care and medical care in rural areas can also have an “adhesive effect”. The evaluation of similar programs has demonstrated a positive effect on readiness to work as a family practitioner (in a rural area) after completing medical studies [11].

The “Landpartie 2.0” program can thus be considered an alternative to the "rural physician quotas" that have recently been introduced by several federal German states. Whereas the "rural physician quota" represents a binding decision to choose to work as a family doctor in a rural area previous to medical studies [24], the priority program described here provides students with the opportunity to try working as a young doctor and to get to know their own personal interests, based on the appeal of working in a rural area, and their own intrinsic motivation. At the same time, they can develop their medical skills and competencies.

Preliminary evaluation results show that the expectations and demands that students wished to see fulfilled by “Landpartie 2.0” were satisfied, and approval of practice internships, the series of seminars and the annual one-day excursion was high to very high. Over the medium- and long-term, evaluative graduate destination surveys will illustrate whether participation in the program increases interest in family medicine. They will also show whether, in comparison to students with similar interests that did not participate in the program, the probability is higher that participants will choose to do a four-month elective in their practical year and/or specialist training in family medicine, and whether they will later decide to work as family doctors (in a rural region). 

The present evaluation takes its place among further investigations that have been conducted into other established programs that are available in Germany. Since 2011, the “Family Medicine Class” has provided 20 medical students with a clinical elective that focuses on primary care. An evaluation after the first two years showed that the acquisition of skills resulting from participation in the program had a positive influence on the decision to select family medicine as a medical specialty [9]. The study of a preclinical elective that focused on family healthcare in a rural area around Leipzig demonstrated that participation in a course on family medicine strengthened the position of family medicine as the first choice among career options [10].

Despite the comprehensive methodological approach employed in the development phase, it is by no means certain that the program developed for “Landpartie 2.0” will awaken the interest of medical students in the future. The question to what extent participation in the program increases the readiness to, for example, complete a four-month elective in the practical year and/or specialize in family medicine with a view to working in a rural area remains uncertain. Amongst other considerations, it should be taken into account that the participating students made up, a priori, a strongly self-selected group. Only students with a basic interest in family medicine, and who are at least prepared to consider specializing in the field and working in a practice outside a large city, are likely to apply to participate in the program. However, other similar programs also face this methodological problem [7], [25]. It is therefore essential to take this selection effect into account in more detailed evaluations.

To the best of our knowledge, the longitudinal priority program presented here is the first in Germany to focus on promoting family medicine in a rural area during medical studies. Similar programs in other regions generally concentrate on family medicine but have no explicit rural focus. The broad methodological approach to the conceptual development of the program enabled best practices, the needs and wishes of students, as well as existing curricular and legal framework conditions in Germany to be taken into account early on in the process. In the development phase, it was therefore clear early on what long-term personnel and financial resources the program would have to mobilize to ensure its implementation was sustainable. Furthermore, the program was designed to be voluntary, available from the first clinical semester onwards, and to focus on practical training as far as possible. 

## Conclusion

The optional “Landpartie 2.0” program was successfully planned and integrated into the clinical phase of medical studies. The chosen methodological approach contributed towards ensuring that the opportunities and limitations of such a program were recognized and considered in its conceptual development. 

Preliminary evaluation results indicate that “Landpartie 2.0” meets the needs of students, makes an important contribution towards improving medical training and, despite its rural focus, also appeals to students from more urban regions. Overall, it is the view of the authors that the longitudinal and comprehensive character of the developed priority program can positively influence the further training and career paths chosen by medical students in favor of family medicine. Participation in a voluntary teaching program that forms an integral part of medical studies is therefore a promising alternative to the so-called “rural physician quota”.

However, any attempt to secure the availability of young professionals in a specific field requires corresponding personnel and financial resources, and these are not (currently) available at a number of locations. The repeatedly affirmed readiness to promote family medicine in medical studies should therefore be followed by concrete steps, and should in particular be aimed at ensuring the sustainable financial footing required to implement the necessary measures. 

## Notes

^1^ The “Landpartie 1.0” permits interested students to complete a block internship in family medicine in rural practices in cooperation with the District of Fulda. It is complemented by special undertakings and offers (Event Day, voucher for leisure activities, reimbursement of costs of accommodation and travel)

^2^ Our intention was that practices should be in communities with fewer than 20,000 inhabitants. With two exceptions (both with 25,000 inhabitants), it was possible to satisfy this criterion. 

## Acknowledgements

We would like to thank all the practices, districts, experts and students that played such an active role in helping us develop “Landpartie 2.0”.

## Funding

This project was supported by local authorities in the districts of Bergstrasse, Fulda and Hochtaunuskreis.

## Competing interests

The authors declare that they have no competing interests. 

## Figures and Tables

**Table 1:**
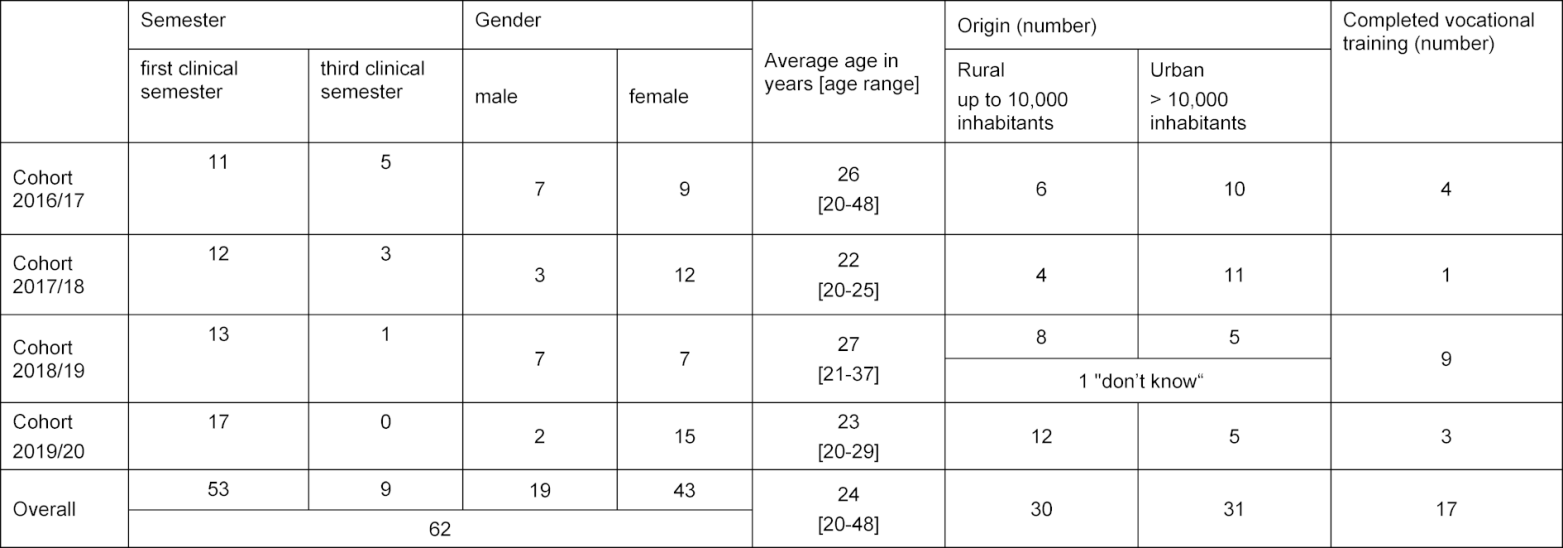
Socio-demographic details at time of application (around November of each year); (Source: proprietary research).
